# The Thirty-Eighth Annual Meeting of the Medical Society of the State of North Carolina

**Published:** 1891-07

**Authors:** 


### THE THIRTY-EIGHTH ANNUAL MEETING OF THE MEDICAL SOCIETY OF THE STATE OF NORTH CAROLINA.

ASHEVILLE, N. C., May 26, 1891.

The thirty-eighth annual meeting of the above Society was called to order in the court-house in this city at 10 o'clock this morning, by Dr. M. H. Fletcher, Chairman of the local Committee of Arrangements. Prayer by the Rev. Charles W. Byrd.

The address of welcome was delivered by Hon. Richmond Pearson, of this city: and was responded to, in behalf of the Society, by Dr. L. Julien Picot.

Dr. Richard H. Lewis, President, then announced the Society as ready to proceed to business, and called for the roll-call. There were present upwards of two hundred and fifty members representing all sections of the State.

After the reading of several invitations to the Society, the President read his annual address.

On motion a committee of three was appointed to consider the salient points in the address and report for the action of the Society. The President appointed on this committee, Drs. W. P. Beall, J. Howell Way and Frank W. Brown.

The orator, Dr. L. G. Broughton, was detained at home on account of sickness, and a message of sympathy was sent him.

Dr. J. W. Long gave some practical demonstrations in Urinalysis.

Dr. Thomas F. Wood referred to the paper he had presented at the last meeting of the Society on Ehrlich's Urinary test for typhoid fever. It was then a new thing and somewhat in the experimental stage. He had sent to several members since that meeting samples of the reagents, with full directions for using, and hoped they would give their experience. He had made a good many laboratory notes which he had inadvertently left behind, but the test was now a well established thing, and he had the greatest confidence in its value in determining a simple from a specific fever. The test as originally given has been somewhat modified by Ehrlich himself; and Dr. Carl Simon, of the John Hopkins University has also modified it. He adds to the specimen of urine to be tested about twice its quantity of absolute alcohol, and to this the solution of sulphanilic acid. If typhoid fever is present the deep eosine color will be developed.

Dr. R. L. Payne, Jr., read a paper on the "Importance of Urinalysis in the Diagnosis of Obscure Diseases."

Dr. Long said that he was in accord with the author in believing that there was no such thing as physiological albuminuria.

Dr. Wood called attention to the marked alteration in the urine of those patients having the febrile symptoms of influenza. The urine was sometimes so offensive that it had to be taken out of the room at once. At first he did not understand the significance of this. Examination showed that in a very large number of cases there is the reaction for indican. This points to the fact that the fever of grip is one of graver import than we would at first suppose, and was one of the reasons for the tardy convalescence.

Dr. Karl Von Ruck read a paper on the "Present Status of Tuberculosis."

In reply to a question Dr. Karl von Ruck said it had been his experience that those patients having a predisposition and suffering with a pulmonary lesion were apt to contract disease if they were exposed to the germs.

Dr. Beall reported success with the treatment suggested by Drs. Gibbes and Shurley, of Detroit—the inhalation of chlorine gas and the injection of iodine and the chloride of gold and sodium. He has used the treatment in eleven cases with gratifying results. The inhalation of chlorine cannot be borne at first, but the patients soon obtain a tolerance.

Dr. J. W. Byers read a paper entitled "Tuberculin and Its Failure."

Dr. Von Ruck discussed Dr. Byers' paper, saying that it was based on theory only, and he thought the author was not up with the literature of the day on the subject. There had been a great many cases of improvement related by reliable authors since the introduction of the remedy, and even in the first two months of its use in Germany, the report of the minister of public instruction shows that in 1,010 cases which have been treated on an average only a little over three weeks, shows a surprising number of cures and improvements. He had shown from a five month's experience in over a thousand injections, given to forty patients,that all but two cases improved, seven have already been discharged apparently cured, and many are greatly improved and their progress is toward a cure.

The Society then went into conjioned meeting with the North Carolina Board of Health ; Dr. H. T. Bahnson, President of the Board of Health, taking the chair.

He lamented the fact that the Board of Health still occupied the anomalous position it did at its incipiency. That as the services of bodies, such as this, that had only advisory powers, were seldom called upon, that therefore the Board had but little progress to report. He called the attention of the Society to the slight that had been shown the Board of Health and the medical profession of the State by the last Legislature, in adopting for use in the public schools a book gotten up by the so-called "Woman's Christian Temperance Union" on the evil effects of alcohol on the human system. He regretted the ignorance of the Legislators who entrusted the mental hygiene of our children to the Secretary of a body like that.

He alluded also to the lack of interest taken by the profession throughout the State in the work of the Board. Also to the backwardness of the people in matters relating to hygiene and the preservation of health.

The Secretary of the Board, Dr. Thomas F. Wood, said he would not detain the Society with a long report, but called their attention to one or two special points in the Third Biennial Report of the Board which was just from the publisher's hands and which he distributed.

He referred to a table showing the present condition of the jails and poorhouses throughout the State as compared with their condition at the time of the organization of the Board. In many instances new jails and poor houses had been built and hospitals established for the care and treatment of the sick and insane.

There was but very few cases of small-pox occured in the State, and the State had placed at the disposal of the Board $2,000 to be used in stamping out any epidemic that threatened to arise from the presence of a single case of any of the more contagious diseases.

Two vacancies occurring on the Board at this meeting, Dr. Thomas F. Wood (re-election) and Dr. S. Westray Battle were elected to fill the vacancies.

Dr. S. D. Booth read a paper on Extra Urine Pregnancy.

As Chairman of the Section on Practice of Medicine, read a paper entitled "Summer Diarrhœa Among Children," and "Some Notes on the Use of Cupric Arsenite in Acute Disturbances of Alimentary Canal."

Dr. J. Howell Way read a paper on "The More General Use of Chloroform in Non-Operative Labor."

Dr. W. P. Whittington presented a paper on "Inflammation of the Uterine Appendages."

Dr. Albert Anderson, Chairman of the Section of Pathology and Microscopy, read his report.

Dr. Daniel Lewis, delegate from the New York State Medical Society, was invited to the privileges of the floor. He said he had been much interested in the discussion. He congratulated the Society in having the control of medical matters in the State, but prophesied that they would not retain their monopoly many years unless they kept a pretty sharp eye on the Legislature. He could congratulate them also on having no medical colleges, for they had found most of the opposition to the establishment of a Board of Medical Examiners to come from their medical colleges.

At the evening session Dr. Thomas E. Anderson, the annual Essayist, read a paper on the "Evolution of Surgery."

Dr. Daniel H. Howell, of Atlanta, corrected the statement of the author of the essay in regard to the discovery of ether, claiming that honor, or rather the distinction of being the first to use it, for a Georgia physician.

It was claimed again the next day, by Dr. F. Peyre Porcher, of Charleston, for a South Carolina physician.

Dr. W. C. Galloway opened the Annual discussion by reading a paper on Perityphlitis.

Dr. Jones thought the oft repeated attacks of colic that are often met were frequently cases of mild perityphlitis ; that it is an obscure disease and the operation for its cure should not be lightly undertaken.

Dr. Williams, in view of the fact that one-third of the human race were at one time or another attacked by this disease in some form, rather discountenanced the promiscuous operations suggested by modern surgeons. Country physicians are not used to abdominal surgery, and cannot put their finger into the cavity and by feeling tell the condition of things in the cavity.

Dr. Bahnson scouted the idea of the great frequency of the disease, seeing that in his 22 years experience he had not met with a case. Suggested as a preventative measure for those who found it occured so often, the removal of the appendix in infants.

Dr. Wood recited several cases coming under his observation in the last twelve months, in which aspiration revealed the presence of a pus cavity wnich was washed out through a canula with recovery of the patients. Thought aspiration was necessary in many instances, as only the ocular demonstration of the pus could convince the friends of the importance of an operation. We should not be guided too blindly by the advice of specialists.

Dr. Weaver presented the following conclusions :

1. Inflammation in the right iliac fossa are due to disease of the appendix.

2. When an abscess forms it is found primarily within the peritoneal cavity, and not in the post-peritoneal tissues.

3. Many mild and some severe cases are cured without operation.

4. When high fever, localized pain and tenderness, with a hard tumor in the right illiac fossa occur, an operation is indicated. It is not wise to aspirate—incision is the best both for exploration and operation.

Dr. Daniel Lewis said that the surgeons of New York were not altogether in accord with Dr. McBurney's conclusions and treatment on this subject. Thought it was a rather rare disease. In 20 years of hospital and private practice he had seen only six cases. The operation had been done a good many times when the operation revealed no evidences of disease of the appendix. In one of these cases at least death resulted. Thought it unwise to recommend operation on all these cases as early as the fifth day; or at all, for nine out of ten go on to suppuration and the pus can be evacuated in the old way.

The following officers were elected for the ensuing year:

President—Dr. W. T. Cheatham, Henderson.

First Vice-President—Dr. Thomas S. Burbank, Wilmington.

Second Vice-President—Dr. J. W. Long, Randleman.

Third Vice-President—Dr. W. H. Cobb, Goldsboro.

Fourth Vice-President—Dr. W. D. Hilliard, Asheville.

Secretary—Dr. J. McHays, Oxford.

Treasurer—Dr. C. M. Poole, Salisbury.

Orator—Dr. J. W. Long.

Essayist—Dr. O. McMillen.

Dr. Hodges asked the Society to take some action in regard to counter prescribing by druggists. It was a very common practice in his town, and especially in the matter of venereal diseases, the druggists did very nearly all the prescribing. According to the laws of the State these druggists were liable to indictment, and their local society had determined to bring the name of any one who gave prescriptions requiring scientific knowledge before the grand jury. He thought some means should be taken to make the people understand the difference between a regularly graduated physician and the druggists who allowed themselves to be called "Doctor."

The president was glad the matter had been brought up. A bill had been introduced by the druggists at the last legislature to prohibit physicians from opening a drug store in any town of more than 800 inhabitants without procuring a license from the Pharmaceutical Association.

Mr. Hancock, the secretary of the North Carolina Pharmaceutical Association, was present, and explained that the Pharmaceutical Association were opposed to prescribing by druggists; that the laws prohibited it, and that he hoped this Society would pass a resolution condemning it.

Dr. McDuffie emphasized Dr. Hodge's remarks, and called attention to the fact that even the little boys in the drug stores were permitted to prescribe for gonorrhœa in the case of boys of their own age who had contracted the disease and were ashamed to go to a physician.

On motion, a committee was appointed to confer with a committee from the Pharmaceutical Association for the purpose of putting an end to this evil.

Dr. W. H. Wilson read a paper as chairman of the section on therapeutics.

Dr. Isaac N. Taylor, chairman of the section on State medicine, read a paper on "The Care of the Insane."

In the discussion that followed, Dr. Murphy, manager of the North Carolina hospital for the insane, at Morganton, cited two instances of great cruelty to the insane patients by the keepers of the poor houses where they were confined.

A committee was appointed to revise the laws of the Society, and suggest a condensed substitute to be presented for the consideration of the next meeting of the Society.

Dr. Robinson, of Goldsboro, made a verbal report of a case of hydrophobia in which the hypodermic injection of antipyrin controlled the spasm in a remarkable manner. The sight of water had thrown the patient into spasms which prevented the taking of any nourishment. The injection reduced the pulse from 156 to 125, and soon after the patient drank two saucers of milk, and still later, quite a quantity of water.

On motion, the Society adjourned to meet in Wilmington on Tuesday, the 31st of May, 1892.

